# Clinicopathologic and survival results in serous endometrium carcinoma and subgroup analysis for mixed serous and pure serous histology

**DOI:** 10.4274/jtgga.2017.0065

**Published:** 2018-03-01

**Authors:** Alpaslan Kaban, Samet Topuz, Hamdullah Sözen, Yağmur Minareci, Yavuz Salihoğlu

**Affiliations:** 1 Department of Obstetrics and Gynecology, İstanbul University İstanbul School of Medicine, İstanbul, Turkey

**Keywords:** Endometrium cancer, histologic type, serous component

## Abstract

**Objective:**

To review the clinicopathologic and survival outcomes of patients with serous endometrial cancer (EC) and to investigate subgroup analysis based on pure serous and mixed serous EC subtypes.

**Material and Methods:**

Patients who underwent EC surgery between 2002 and 2014 and who were reported as serous EC were enrolled in the study. All patients were diagnosed as having serous EC or mixed serous EC with serous component higher than 10% based on the postoperative pathology report.

**Results:**

A total of 93 patients were analyzed. The median disease-free and overall survival (OS) durations were 49.6 and 32.2 months, respectively. Forty-three patients (46.2%) relapsed and 35 patients (37.6%) died. The histologic type was pure serous EC in 52 (55.9%) and mixed EC in 41 (44.9%) patients. There was no statistical difference between the pure serous and mixed serous groups in terms of age, International Federation of Gynecology and Obstetrics stage, lymphadenectomy, lymph node metastasis or adjuvant therapy combinations. Twenty-nine (55.8%) patients in the pure serous group and 14 (34.1%) in the mixed serous group hade recurrence (p=0.038). Twenty-five (48.1%) patients in the pure serous group and 10 (24.4%) in the mixed serous group died (p=0.034). In the pure serous group, the mean disease-free and OS durations were shorter than in the mixed serous group (59 vs. 81 months and 73 vs. 95 months, log-rank p=0.055 and 0.041, respectively). Histologic type was a significant prognostic factor on recurrence and OS in the univariate analysis (Hazard ratio: 2.404, 95% Confidence interval: 1.01-5.71; 2.027, respectively), but not in the multivariate analysis, which included disease stage and age of the patients.

**Conclusion:**

Compared with pure serous and mixed serous endometrium cancer groups, primary surgical treatments, clinicopathologic features and adjuvant treatments were similar, but there was a survival difference. Patients with pure serous cancer had a worse prognosis. However histology was not an independent factor for survival.

## Introduction

Among the endometrial cancer (EC) subtypes, serous endometrial carcinomas are known as high-risk carcinomas. They account for nearly 10% of all ECs; however, the highest mortality due to EC is seen with serous carcinoma among all subtypes (1,2). Serous EC may either be seen as pure serous carcinoma or together with endometrioid (most common), clear cell or sarcomatous components (3). In this group known as mixed ECs, any component higher than 10% but less than 90% is considered widespread (4). The most frequent is the combination of serous and endometrioid components, which account for less than 1% of all patients with EC (5).

In general, pure serous and mixed serous EC are considered similar in terms of clinical features and survival, and they are treated in similar ways (6). A limited number of studies have investigated whether there was any difference between the two subtypes in terms of clinicopathologic features and survival rates. Survival appears to be similar in a few studies with small sample sizes, especially from pathology departments (3,6). The English literature comprises only a single study with a large sample size comparing the two subtypes one-to-one; in that study, Roelofsen et al. (7) demonstrated poorer survival in pure serous EC as compared with mixed serous EC and proposed that they might be two different entities. These tumors represent an infrequent subset of patients with EC; hence, most studies are retrospective and included a limited number of patients.

Our purpose in planning this study was to review the clinical features and survival results of serous endometrium cancers and to compare them with simple pure serous and mixed serous subgroups based on clinicopathologic features, surgery treatments, adjuvant treatments, recurrence rates, overall survival (OS) durations, and disease-free survival (DFS) durations.

## Material and Methods

Study data were derived from the hospital records of patients who underwent surgery for EC between 2002 and 2014 at the same center. A total of 103 patients who were diagnosed as having pure serous EC or mixed serous EC based on the postoperative pathology report were recruited. Endometrioid type EC with serous component between 10-90% was considered mixed serous EC. The amount of serous component, which was between 10 and 80%, was not taken into account (no data are demonstrated). All patients who fulfilled the definition of mixed serous carcinoma were allocated to the same group. Mixed carcinomas without serous component were not included in the study. All pathologic examinations were performed by the same team of gynecopathologists.

Four patients with unavailable follow-up information, four patients who died of other reasons (2 cardiovascular reasons, 1 pulmonary embolism after femur fracture, 1 during postoperative chemotherapy), and two patients who underwent surgical procedures after neoadjuvant chemotherapy were excluded from the study. Demographic, clinical, surgical and pathological outcomes and data from adjuvant therapy of the remaining 93 patients were recorded on Excel software. Pure serous EC and mixed serous EC groups were compared in terms of clinical features, pathologic features, OS, and DFS.

Permission of the local ethics committee was not sought because this study was planned as a retrospective review. However, all patients signed an informed consent form, which allowed our center to use their clinical data for scientific trials.

### Statistical analysis

Number Cruncher Statistical System 2007 (Kaysville, Utah, USA) program was used for statistical analyses. In addition to the descriptive statistics (mean, standard deviation, median, frequency, ratio, minimum and maximum) used to evaluate the study data, paired group comparison of the quantitative data was made using Student’s t-test for parameters showing normal distribution and the Mann-Whitney U test for parameters not showing normal distribution. Qualitative data were compared using Pearson’s chi-square test, the Fisher-Freeman-Halton test, Fisher’s exact test, and Yates’s continuity correction test (Yates’s corrected chi-square). Kaplan-Meier survival analysis and the log-rank test were used to assess survival. Univariate and multivariate cox analysis were used in the analysis of OS and recurrence-related factors.

## Results

The total number of patients was 93. The demographic and clinicopathologic characteristics of the 93 patients are presented in Table 1. The median OS was 49.6 months (range, 3-120 months), and the median DFS was 32.45 months (range, 2-120 months). Disease recurred in 43 patients (46.2%), and 35 patients (37.6%) died. The histology of tumor was pure serous in 52 (55.9%) patients and mixed serous in 41 (44.9%). Early-stage disease according to the International Federation of Obstetrics and Gynecology (FIGO) (FIGO 1-2) was found in 69.9% of patients and advanced-stage (FIGO 3-4) disease in 30.1% of patients.

**Figure 1:**
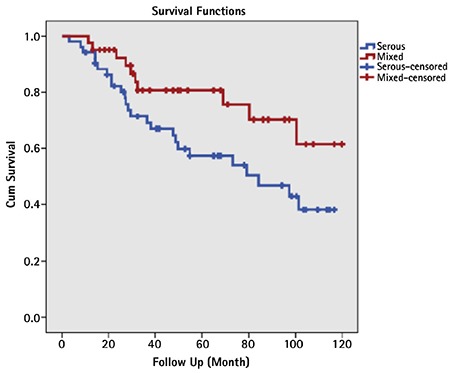
Overall survival graphs of pure serous and mixed serous groups

**Figure 2:**
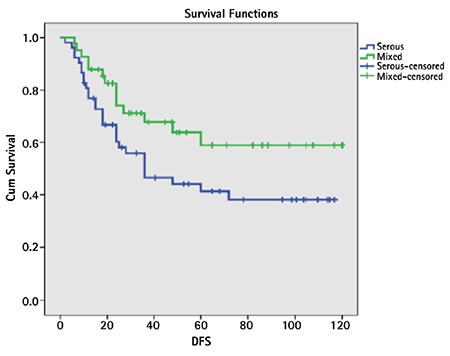
Disease-free survival in the pure serous and mixed serous groups

**Comparative analysis between pure serous and mixed serous groups (Table 2): **There was no statistically significant difference between the two groups in terms of age, parity, chronic disease (diabetes or hypertension), positive peritoneal lavage fluid, endocervical invasion, lymphovascular invasion, depth myometrial invasion, tumor size (cm), and FIGO stage. The groups were not different in terms of the tumor spread, such as presence of tumor in the perimetrium, ovaries, fallopian tubes, omentum, and colon. There was also no statistically significant difference between the groups in terms of the number of patients who underwent lymphadenectomy, the number of lymph nodes removed, the rate of lymph node metastasis, or adjuvant therapy combinations (Table 2, 3). Recurrence was observed in 29 (55.8%) patients in the pure serous group and in 14 (34.1%) in the mixed serous group; the difference was statistically significant (Yates’s continuity test p=0.038). Twenty-five (48.1%) patients in the pure serous group and 10 (24.4%) in the mixed serous group died; the difference was statistically significant (Yates’ continuity test p=0.034). In the serous group, the mean DFS and OS durations were shorter than in the mixed serous group (59 vs. 81 months and 73 vs. 95 months, log-rank p=0.055 and 0.041, respectively) (Figure 1, 2).

**Univariate and multivariate analysis of overall survival and recurrence-related factors (Table 4, 5):** In the univariate analysis, subtype and stage were significant factors for recurrence and OS but not lymphovascular invasion. Significant factors were included in the age-adjusted multivariate analysis. Age and FIGO stage were independent prognostic factors but not subtype in multivariate analyses.

## Discussion

Serous endometrial carcinoma was first described by Hendrickson et al. (8) as tumors histologically similar to ovarian serous tumors and as a different and aggressive subtype of ECs. Patients with serous carcinoma represent 10% of ECs but are responsible for 39% of EC deaths (1). In the literature, data about survival in serous endometrial carcinomas are quite different. In a review, recurrence rates in stage I were reported as 7-50% (9). In the present study, 46.2% of patients had disease recurrence.

We investigated patients with serous endometrial carcinoma (serous component ratio at least 10%). In fact, this study focused on whether there was a difference in the clinicopathology and survival in patients with mixed serous and pure serous carcinoma. Mixed serous-type ECs are relatively less understood; the literature comprises fewer publications, and they are less prevalent. In a study conducted by the Gynecologic Oncology Group, Brinton et al. (5) detected mixed serous/endometrioid histology in 26 (0.6%) of 3828 patients with EC.

In the present study, we investigated patients with pure serous endometrial carcinoma and patients with mixed serous endometrial carcinoma with serous component of more than 10%. The clinicopathologic and survival parameters were statistically compared between these two subtypes.

The FIGO stage, which is considered as the most critical factor determining survival, was statistically similar in both groups (p=0.195). In more detail, we examined the spread of the disease via detailed analyses and determined no significant difference between the groups in terms of involvement of the perimetrium, fallopian tubes, intraperitoneal fluid, intestine or omentum (Table 2). Likewise, the number of patients who underwent lymphadenectomy, the number of patients with lymph node metastasis, and the number of lymph nodes removed were similar between the groups (Table 2). The groups were also similar in terms of the depth of myometrial invasion, endocervical involvement, tumor size, and lymphovascular space invasion, which are given as poor prognostic factors (Table 2).

Reviewing adjuvant therapy options in detail, we see seven different combinations of chemotherapy, external beam radiation therapy, and brachytherapy. Therapy options were statistically similar in the groups (Table 3).

Despite the absence of a difference between the groups in terms of either clinicopathologic findings or stage or treatment approach, the pure serous group seems to have had a worse prognosis. However, in the multivariate analysis, histologic subtype was not an independent factor for recurrence or survival (Table 4, 5). In a study on this topic, Roelofsen et al. (7) compared these two subtypes and found a 2.9-fold greater risk for recurrence and a 2.6-fold higher risk of death in the pure serous subtype. The authors suspected the two subtypes might be two different entities. To the best of our knowledge, no other clinical studies have compared these two groups.

The primary and adjuvant treatment of mixed serous and pure serous histology is not different. The European Society for Radiotherapy & Oncology, the European Society of Gynaecological Oncology, and the European Society of Medical Oncology in the first meeting reported that hysterectomy and bilateral salpingo-oophorectomy was the mainstay of therapy in apparent stage I disease and that radical hysterectomy was not recommended in stage II disease, whereas complete cytoreduction was required in advanced disease stages (10). However, there is no documentation on ovarian preservation. Bilateral salpingo-oophorectomy is mandatory (10). Differently, in the multidisciplinary panel, the authors stated staging omentectomy should be considered in serous carcinoma but not in clear cell or undifferentiated endometrial carcinoma and carcinosarcoma. No specific recommendations for mixed serous types have been provided. In adjuvant treatment issue, the largest retrospective study conducted to date suggested a survival benefit for the combination of chemotherapy and radiotherapy in uterine serous cancer (11). In the present study, adjuvant treatment combinations were similar for pure serous and mixed serous groups. In our clinic, an adjuvant treatment was given to all patients with non-ECC and the adjuvant treatment approach is not different for mixed serous or pure serous histologies.

The molecular biology and cellular origins of mixed-type endometrial carcinomas are poorly understood. In a study, molecular analysis of these two subtypes revealed that they were close in terms of molecular features and suggested that they could be treated similarly (12). In the present study, the groups were treated in similar ways in terms of both primary surgical therapy and adjuvant therapy. The pathologic outcomes of the groups were also similar (Table 2, 3). However, relapse and death rates were not similar.

It is arguable that planning more aggressive adjuvant therapy in a patient group where recurrence is considered to be more frequent — or contrarily, avoiding aggressive adjuvant therapy in a group with better prognosis — may be more convenient. However, randomized controlled studies are required to recommend different treatment to these groups.

Why survival is better in the togetherness of serous and endometrioid components, what kind of interaction influencing survival exists between two components, which of the components exists first in the endometrial cavity and why the other is included thereafter and whether this is important, and detailed molecular biology and cellular origins of these two subtypes will be clarified in future studies.

This study has several limitations; the retrospective design and relatively small sample size for comparing the effect of adjuvant treatment options are the main limitations of the study. Also, it might be better to evaluate clinical outcomes according to serous component rates, but dividing the mixed histology into subgroups will reduce sample size and the serous component ratio of some patients was not clear. However, mixed serous histology (serous plus endometrioid) is not a common clinical entity (5).

In conclusion, this trial supported that mixed serous carcinomas have significantly better prognoses than pure serous carcinomas. However, histologic subtype was not an independent prognostic factor. Studies with large numbers of patients or multicenter studies that support our results may help in making a clearer picture.

## Figures and Tables

**Table 1:**
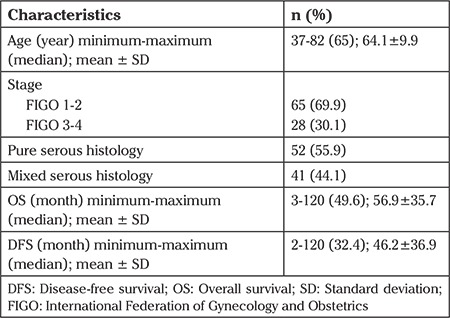
Stage, histology and survival outcomes of ninety-three patients

**Table 2:**
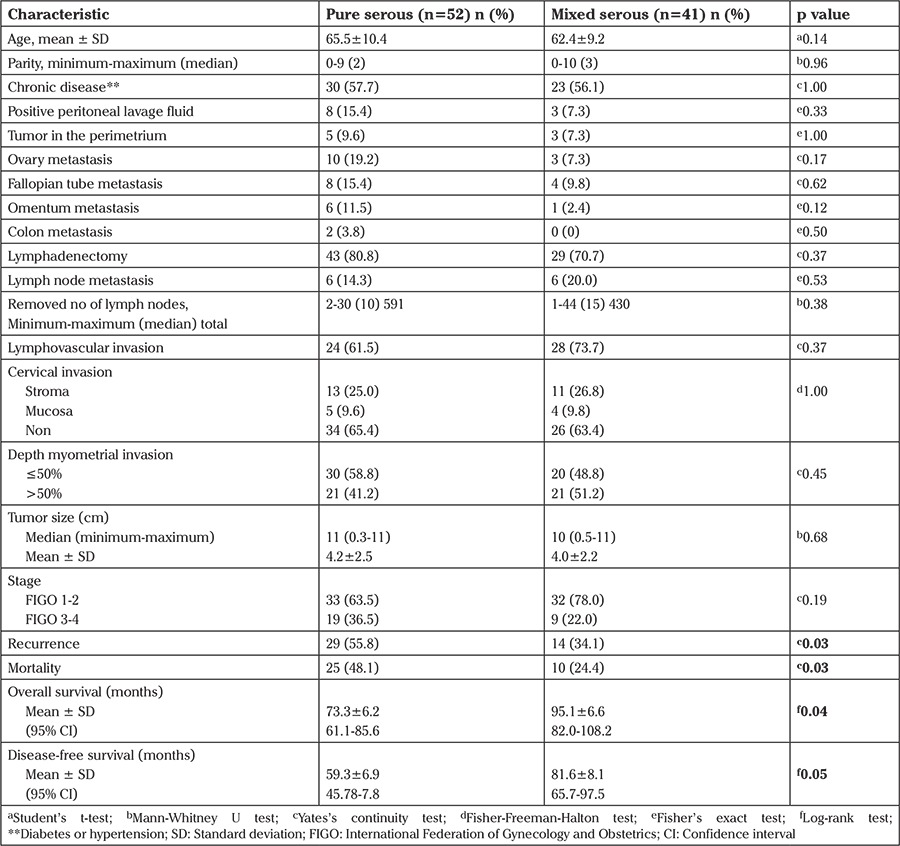
Comparison of clinicopathologic and survival features between the serous and mixed carcinoma groups

**Table 3:**
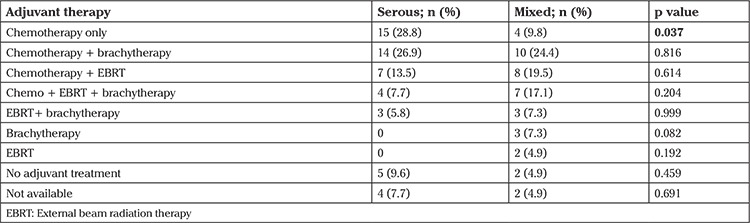
Adjuvant therapy combinations

**Table 4:**
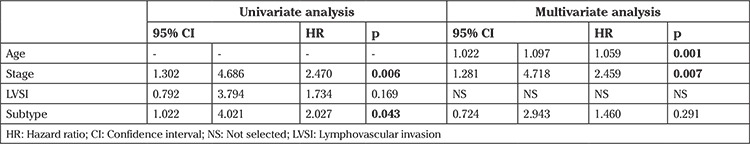
Univariate and multivariate analysis of effective factors for overall survival duration of the ninety-three patients

**Table 5:**
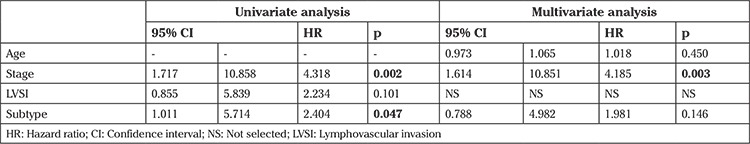
Factors associated with recurrence by univariate and multivariate analysis
